# Using artificial intelligence based language interpretation in non-urgent paediatric emergency consultations: a clinical performance test and legal evaluation

**DOI:** 10.1186/s12913-025-12263-1

**Published:** 2025-01-24

**Authors:** Julia Brandenberger, Ian Stedman, Noah Stancati, Karen Sappleton, Sarathy Kanathasan, Jabeen Fayyaz, Devin Singh

**Affiliations:** 1 Division of Paediatric Emergency Medicine, Department of Paediatrics, Inselspital, Bern University Hospital, University of Bern, Bern, Switzerland; 2 Division of Pediatric Emergency Medicine, Hospital for Sick Children, Toronto, Canada; 3 Edwin S.H. Leong Centre for Healthy Children, University of Toronto, Toronto, Canada; 4 Child Health Evaluative Sciences, SickKids Research Institute, Toronto, Canada; 5 School of Public Policy and Administration, York University, Toronto, Canada; 6 Language Interpretation Services, Hospital for Sick Children, Toronto, Canada

**Keywords:** Pediatric emergency medicine, Artificial intelligence, Minor health visits, Non-urgent health visits, Pediatric migrant health, Pediatrics

## Abstract

**Objective:**

To evaluate the accuracy of Google Translate (GT) in translating low-acuity paediatric emergency consultations involving respiratory symptoms and fever, and to examine legal and policy implications of using AI-based language interpretation in healthcare.

**Methods:**

Based on the methodology used for conducting language performance testing routinely at the Interpreter Services Department of the Hospital for Sick Children, clinical performance testing was completed using a paediatric emergency scenario (child with respiratory illness and fever) on five languages: Spanish, French, Urdu, Arabic, and Mandarin. The study focused on GT's translation accuracy and a legal and policy evaluation regarding AI-based interpretation in healthcare was conducted by legal scholars.

**Results:**

GT demonstrated strong translation performance, with accuracy rates from 83.5% in Urdu to 95.4% in French. Challenges included dialect sensitivity and pronoun misinterpretations. Legal evaluation indicated inconsistent access to language interpretation services across healthcare jurisdictions and potential risks involving data privacy, consent, and malpractice when using AI-based translation tools.

**Conclusions:**

Google Translate can effectively support communication in specific non-critical paediatric emergency scenarios. However, its use necessitates careful monitoring, understanding of its limitations, and attention to dialect and literal translation risks along with equity considerations. Establishing legal and policy frameworks for language interpretation in healthcare is crucial, alongside addressing funding and data security concerns, to optimize the use of AI-based translation tools in healthcare contexts.

**Supplementary Information:**

The online version contains supplementary material available at 10.1186/s12913-025-12263-1.

## Background

Effective communication between patients and health care providers is a prerequisite for safe medical care [1]. In diversity-rich settings where many patients speak different languages than those spoken at the local facilities, communication is considered a key challenge [2]. Previous studies have shown important inequities for patients exclusively fluent in non-local languages [3, 4]. Although the use of professional language interpretation in health encounters is widely recognized as best practice and in some countries also required by law, evidence shows a significant underuse [5–8]. A study conducted at a paediatric emergency department described that professional language interpretation was only used for 15% of patients not speaking local languages, despite 24 h phone interpretation being available [9]. Explanations for the underuse included: no systematic screening for language proficiency of patients, low patient awareness of their own right to request access to translation services, health care providers with limited training on transcultural competencies and time constraints [10–12]. In many health care settings, no funding is available for professional interpretation services, making it practically impossible to access language assistance. One potential solution is artificial intelligence (AI) based language interpretation [13]. In contrast to in-person/phone interpretation, many applications like GoogleTranslate [14] are immediately available at any time of the day in multiple languages without significant cost or logistical preparation. This might be one reason why they are already unofficially used in many health care facilities [15]. Although promising performance was described for AI-based written translations of scientific abstracts [16] and emergency department discharge papers [17], their performance showed ambiguous results for live, oral interpretations during health encounters [18, 19] and low performance for translations of pharmaceutical instructions [20]. AI-based applications have not been sufficiently tested or legally evaluated, leaving patients and providers in a situation of uncertainty about the quality of language interpretation and potential legal implications if problems occur [18, 21]. These concerns gain importance in regard to the recent, exponential rise of large language models which can also provide language interpretation [22]. This study aimed to shed light on the safety of the unofficial use of AI-based language interpretation and test the performance across multiple languages via a simulated low-acuity paediatric emergency case. We also aimed to determine the legal issues that might arise when using AI-based interpretation devices in healthcare.

## Methods

Research methodology/reporting was guided by the Standards for Reporting Qualitative Research (SRQR) [21] and the COREQ criteria [23].

One AI-based hardware device and two AI-based mobile applications were assessed based on their published performance in the most frequently needed languages at [Hospital Name], in [City, Country]. As google translate (GT) showed the most promising results in a language performance screening, provided the best customer support, was free of charge and commonly utilized it was selected for this study.

Five paediatric non-urgent scenarios were developed by two paediatric emergency physicians and pilot-tested. The study team agreed on the scenario with a respiratory tract infection (Supp. data 1) and fever as the most common and relevant low-acuity paediatric emergency presentation and chose it for the in-depth performance testing.

### Clinical performance testing

**Fig. 1:**
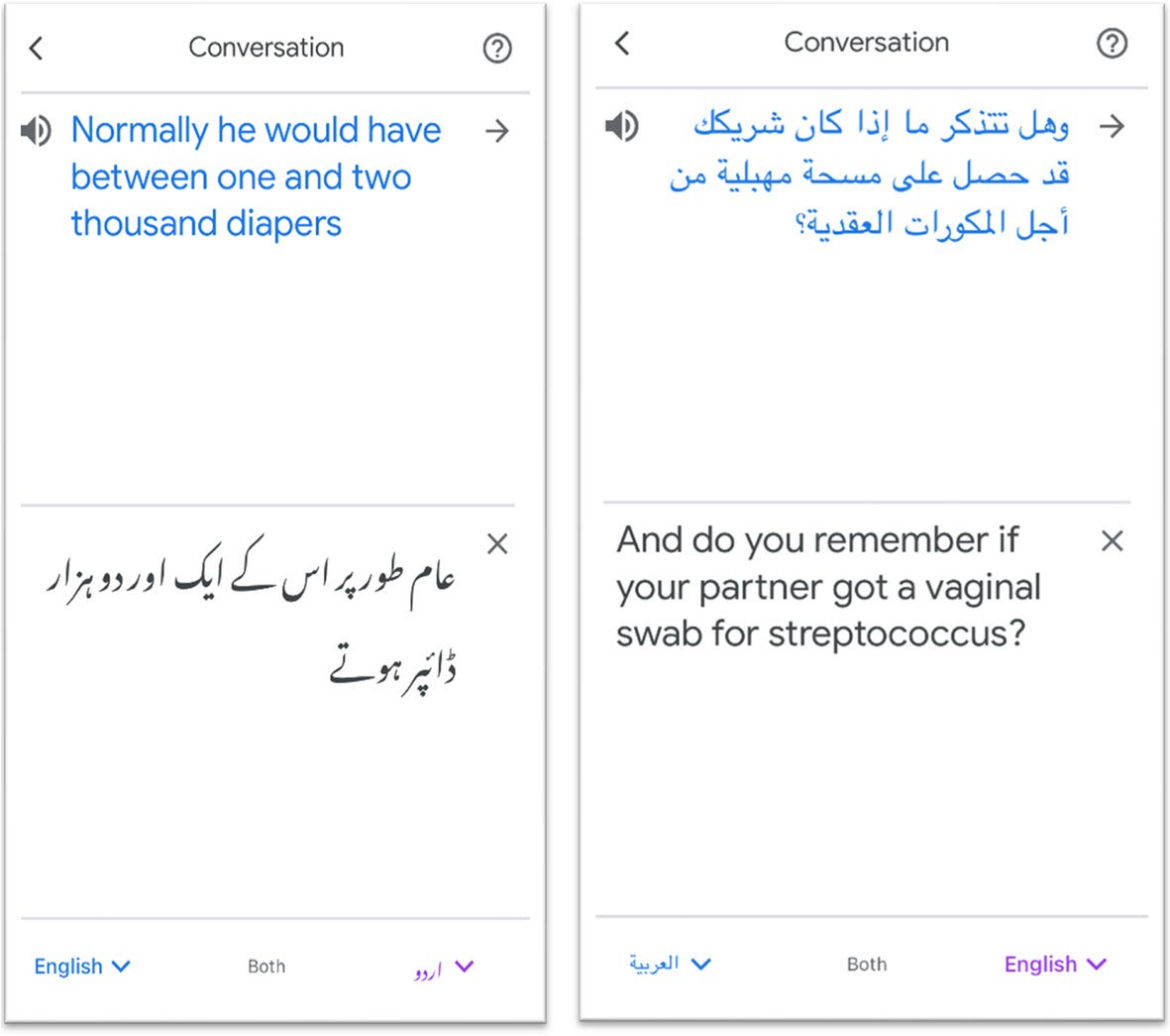
GT conversation mode, depicting an incorrect (1a: Urdu to English) and a correct (1b: English to Arabic) interpretation

Based on the methodology used for language performance testing conducted at the Interpreter Services Department at The Hospital for Sick Children (Toronto), an evaluation tool was developed, tested and modified by an interdisciplinary team of professional interpreters and clinicians (Supp. data 1). Tests were conducted in the five most frequently needed languages at the hospital namely Spanish, French, Urdu, Arabic, and Mandarin using Google Translate (GT) version 6.55.0. Professional interpreters and clinicians fluent in one of these languages and English were selected based on the following criteria: 1) participants needed to speak the language other than English on the level of a native speaker (self-reported by the clinicians and cross-checked by the research team prior to recruitment) 2) They needed to have lived and worked at health facilities in a country where the language is predominantly spoken. A professional interpreter acting as caregiver in the non-English language and a clinician, fluent in the tested language acting as an English-speaking physician, simulated the clinical scenario and evaluated GT. Moderation of the performance testing was done by JB (female, MD-PhD, pediatrician-researcher). Her tasks included to explain the study and ensure coherence in the evaluation of GT performance across different languages. JB also documented ad-hoc discussions between interpreters and clinicians, later serving as qualitative material to provide in-depth insights into the performance test. JB did not interfere directly into the conversation between study participants. The conversation mode of GT was used (Fig. 1).

GT provided a real time transcript of the source language spoken (source language-transcript) and a transcript of the interpretation in the target language (target language-transcript). GT then verbalized the interpretation in the target language via the mobile phone speaker. If a mistake was detectable in the source language transcript, the participant stopped the interpretation and repeated the sentence maximally twice. If the application could not correct the mistake, the paragraph was categorized as an error detected but not corrected. If a mistake was not detectable in the source language transcript and led to a misinterpretation, it was classified as an undetectable misinterpretation. The primary outcome parameter was defined as the sum of paragraphs that were correctly interpreted plus those where mistakes were corrected in a maximum of 2 attempts. All sessions were started with a 15 min introduction to establish a relationship, communicate the study goals and the researchers background. Data was reviewed by participants and stored in excel files at work. Free-text notes were part of the results and summarized by languages, codes, and overarching categories using a step-by-step approach inspired by the principles of qualitative content analysis [24].

### Legal/policy evaluation

A policy survey and a content analysis of legal cases and law/policy publications which discussed the use of AI-based language interpretation in health care settings was conducted by a lawyer with extensive legislative experience in the research context. Identified cases, publications and frameworks were evaluated for their potential to facilitate or limit the use of AI-based language interpretation. Overarching legal/policy concerns and responsibilities were summarized from the perspective of patients, providers, and health care facilities.

## Results

### Overall clinical performance

All 5 performance tests were conducted between February 3^d^ and April 4th, 2023, taking around 180 min each. Overall, GT had strong performance with the final proportion of correctly interpreted paragraphs ranging from 83.5% (Urdu) to 95.4% (French). The proportion of correctly interpreted paragraphs in the first attempt ranged from 76.1% (Spanish) to 91.7% (Arabic, Table 1, Fig. 1).

**Table 1:** Clinical performance summary of Google Translate

Language	Spanish	French	Urdu	Arabic	Mandarin
Ommission of word(s)	2 (1.8)	2 (1.8)	1 (0.9)	0 (0.0)	0 (0.0)
Addition of word(s)	1 (0.9)	8 (7.3)	5 (4.6)	0 (0.0)	1 (0.9)
Word(s) left in source language	0 (0)	0 (0)	2 (1.8)	0 (0.0)	0 (0.0)
Registration of different word(s)	25 (22.9)	16 (14.7)	16 (14.7)	5 (4.6)	7 (6.4)
Partial misinterpretation of paragraph	13 (11.9)	2 (1.8)	6 (5.5)	1 (0.9)	9 (8.3)
Complete misinterpretation of paragraph	12 (11.0)	11 (10.1)	15 (13.8)	8 (7.3)	7 (6.4)
Wrong punctuation leading to misinterpretation	1 (0.9)	1 (0.9)	1 (0.9)	0 (0.0)	1 (0.9)
Total of misinterpretations	26 (23.9)	15 (13.8)	22 (20.2)	9 (8.3)	17 (15.6)
**Identification and correction of misinterpretation**					
Misinterpretation caught and corrected	19 (17.4)	10 (9.2)	4 (3.7)	3 (2.8)	3 (2.8)
Misinterpretation caught but not corrected	3 (2.8)	3 (2.8)	7 (6.4)	1 (0.9)	2 (1.8)
Misinterpretation not caught	4 (3.7)	2 (1.8)	11 (10.1)	5 (4.6)	12 (11.0)

*n* number of paragraphs with mistakes %: proportion out of 109 paragraphs with mistakes; *performance tested for official languages: Chinese mainland (written source language transcript simplified Chinese) and fussah (formal arabic)

Interpretations were more accurate from English to the target languages than from non-English languages to English. In all languages, GT was highly sensitive to dialects and its performance depended on the ability of speakers to adhere to the official language pronunciation. This was particularly important in Mandarin and Arabic: mainland Chinese and Fussah (formal Arabic) were well interpreted whereas when speaking a Chinese dialect or one of the 14 Arabic dialects, performance was lower. Interpretations were also better in paragraphs that were neither too long nor too short, allowing the application to digest the input but also put the single words into context. In all languages, but most in Urdu, Spanish and Arabic, pronouns (he/she and it) were often misinterpreted. Frequently, male gender was used as default. Interpretations also consistently used informal language and addressed subjects on first-name terms.

### Types of mistakes

Omissions, additions, and words that were left in the source language were mistakes rarely made by the application. Registrations of wrong words were more common, particularly in the Roman languages (French and Spanish). All types of mistakes were more likely to lead to misinterpretations if they were affecting words central to the meaning of the paragraph, completely changed the words’ meaning, or affected multiple words in a row. For example, the French to English interpretation of “this should calm him down” remained the same even if the tense of the verb was registered incorrectly in French (cela “devait” instead of “devrait” le calmer). However, it led to a complete misinterpretation of a paragraph in French when the English words “little one” were registered as “Italy won”. Misinterpretations of this nature, especially if they are of a medical term, could have extremely negative impacts on patient experience and even patient safety.

### Identified mistakes leading to misinterpretations

**Fig. 2:**
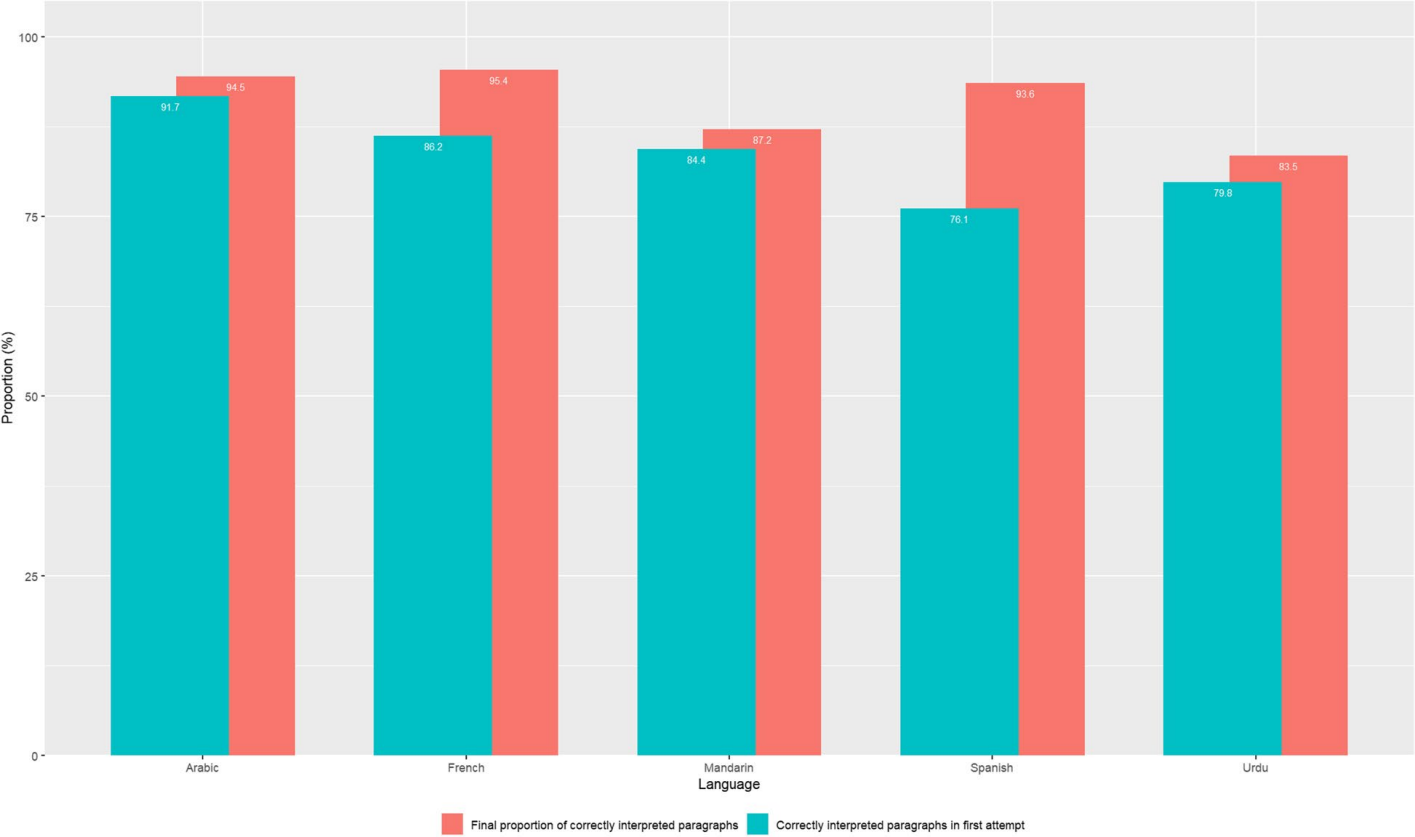
Proportion of paragraphs correctly interpreted by Google Translate in the 5 tested languages

The continuous real-time check of the source language transcript by both speakers (clinician and patient) allowed for the detection of all misinterpretations that were based on registrations of wrong words or misunderstood syntax in the language of the speaker. Frequently, these mistakes were due to the dialect of the speaker or a slightly unclear pronunciation, and improved over the course of the performance test. The real-time check and repetition of the paragraph if a mistake was detected increased the amount of correctly interpreted paragraphs per language by 2.8% (Mandarin) to 17.5% (Spanish; Table 1, Fig. 2). Once a mistake leading to a misinterpretation was detected, only one (0.9%; Arabic) to 7 paragraphs (6.4%; Urdu) remained uncorrected after the maximum of 2 additional attempts, highlighting the overall strong performance of GT.

### Unidentified mistakes leading to misinterpretations

In Spanish, most of the misinterpretations that were not caught by the source language transcript originated from the synonymous use of “si” (meaning “yes” and “if” depending on the context). If the application chose the wrong synonym in the target language, the meaning of the message was frequently not delivered. In French, unidentified misinterpretations were very rare, however important. The sentence “il est vraiment enrhumé” (he’s pretty congested) was interpreted as “he is really pissed off”. The interpretation was correct if the adverb (vraiment) was left out. In Arabic, Urdu and Mandarin, unidentified misinterpretations where more common. In all 3 languages, “congestion” (runny nose) was interpreted as “traffic jam” and the height of the fever was interpreted as “tall”. In Urdu and Mandarin, the verb “drinking” was misinterpreted as “drinking alcohol” whereas the interpretation was correct if the verb “feeding” was used. Potentially dangerous misinterpretations included misinterpretations of numbers in Urdu. While the Urdu source language transcript was correct, the Urdu-sentence “normally he would have had like 2 or 3 extra (diapers)” was interpreted to English as “normally he would have between one and 2000 diapers.” (Fig. 1a). The height of the fever was also only interpreted correctly, if English numbers and decimals were used by the Urdu-caregiver – a practice that, according to the Pakistani interpreter and MD, is used by some but not all families. Difficulty with interpreting numbers correctly existed also in Mandarin. Particularly if a Taiwanese dialect was spoken, 4 and 10 were at risk to be misinterpreted. As Mandarin has a very different structure of describing the past, present and future, mistakes in the times happened occasionally, potentially leading to misinterpretations. The application made relatively few misinterpretations in Arabic, if Fussah was spoken and common language was used. As in all languages, interpretations were very literal and sometimes lead to misinterpretations: the specific meaning of “term” in “term baby” was misinterpreted as the literate interpretation of “term” as “word”, becoming “word baby”. However, some sentences where mistakes were common in other languages were well-interpreted in Arabic (Fig. 1b).

### Misinterpretations by language category

While slang words were well interpreted in most languages, the interpretation of medical terms was more prone to mistakes and varied between languages (Table 2).

**Table 2:** Misinterpretation by language category (lay/medical/slang)

Language	Spanish	French	Urdu	Arabic	Mandarin
**Paragraphs including slang (*n* = 8)**	n (%)	n (%)	n (%)	n (%)	n (%)
Registration of different word	2 (25.0)	0 (0.0)	1 (12.5)	0 (0.0)	0 (0.0)
Misinterpretation	2 (25.0)	0 (0.0)	1 (12.5)	0 (0.0)	0 (0.0)
Misinterpretations caught	1 (12.5)	0 (0.0)	1 (12.5)	0 (0.0)	0 (0.0)
**Paragraphs using common language (*n* = 81)**					
Registration of different word	17 (21.0)	12 (14.8)	8 (9.9)	4 (4.9)	6 (7.4)
Misinterpretation	18 (22.2)	12 (14.8)	11 (13.6)	5 (6.2)	11 (13.6)
Misinterpretations caught	16 (19.8)	11 (13.6)	6 (7.4)	3 (3.7)	4 (4.9)
**Paragraphs including medical terms (*n* = 20)**					
Registration of different word	6 (30.0)	4 (20.0)	7 (35.0)	1 (5.0)	1 (5.0)
Misinterpretation	6 (30.0)	3 (15.0)	10 (50.0)	4 (20.0)	6 (30.0)
Misinterpretations caught	5 (25.0)	2 (10.0)	4 (20.0)	1 (5.0)	1 (5.0)

*n* numbers of paragraphs wrongly interpreted; %: proportion of language subcategory that was wrongly interpreted

As there is no word for crackles or wheezes in Mandarin or Urdu, the application mimicked the sounds instead, potentially confusing or startling parents by the sudden sound output from GT. In Mandarin, Spanish, French and Arabic some other common medical terms like COVID or bronchiolitis were well interpreted. In Urdu many medical terms do not exist and English terms are used instead, which complicated their interpretation for the application as it consequently aimed to interpret every word. The professional interpreter and clinician also described a cultural reluctance to speak about sex/reproductive organs in Urdu, which created an additional challenge. As there is no commonly used term for “vagina” in Urdu, the application used a very poetic term (“the channel where the menstrual blood flows”) which would hardly be understood by many parents.

### Legal/policy evaluation

From a policy perspective, the jurisdictional survey revealed that access to language interpretation in healthcare was inconsistent between both sites and jurisdictions, as well as being under-funded by both public and private payment mechanisms. The content analysis uncovered various legal concerns that accompany the use of AI-based interpretation tools, including the paucity of legal mechanisms (e.g., human rights and other laws) available for patients and families to assert a right to access adequate and timely interpretation. Data privacy and security risks were also identified, as well as the risk of medical malpractice for physicians due to over-reliance on poorly validated translations (Table 3).

**Table 3:** Legal considerations regarding the use of AI-based language interpretation

	**Data Privacy**	**Data Security**	**Consent for use of GT**	**Avoid causing harm due to use of GT**
Patient or substitute decision maker	Ask provider whether, where and how your personal health information (PHI) is being transferred and/or shared	Ask provider whether and how your (PHI) is protected/secured	Make sure you understand the risks and benefits of using GT prior to providing consent	Request an alterative form of translation services if discomfort or concerns with using GT
Healthcare (HC) facility	Unapproved / unsupported use of GT *could* indicate that providers are insufficiently resourced, possibly giving rise to vicarious liability for privacy breach. If this tool is being used unofficially, HC facility should revisit its language translation policies and, to mitigate risk, educate providers about how to protect privacy of PHI and develop pathways for safe utilization of GT as appropriate	If ongoing use of tool is anticipated, HC facility should ensure providers have access to a private version of GT that is housed in architecture meeting or exceeding data security standards	Providers should be trained to understand the risks and benefits of using GT and be able to explain these to a patient in order to obtain informed consent. Alternative translation options should be made available for patients who decline use of GT and require language translation in order to receive healthcare services	Ensure that providers are not forced to use GT due to inadequate access to other translation services. Alternative language translation services should be made available for scenarios where patients cannot adequately use GT safely, decline to consent to GT use, or it is not clinically appropriate
HC provider	Minimize risk by ensuring GT does not collect PHI If PHI will be collected and/or transferred, understand how, why and for what downstream use, if any Seek advice to ensure compliance with relevant privacy laws and local best practice guidelines	First, minimize risk by ensuring GT does not collect PHI If PHI will be transferred, understand whether, how and where it will be stored and how it is protected (i.e. data encryption etc.) Seek advice to ensure data is adequately secured	Make sure patients understand any risks and benefits related to data privacy, security, and possible risks associated with inaccurate translation Explain alternatives to use of GT, even if none available	Ideal if tool has received regulatory approval for intended use as required by local laws Accuracy of tool for intended use case(s) must be adequately validated to the standard that a reasonable physician in similar circumstances with similar resources would agree that its use is appropriate

### No gold standard for language interpretation in health care settings and underfunding

The Canadian Charter of Rights establishes a right to an interpreter during legal proceedings, but not in healthcare encounters [25]. In fact, the Canadian Supreme Court has explained that having public healthcare does not mean all products and services must be covered [26]. This leaves provinces/territories, municipalities or facilities to take on the “budgetary burden” [27] of implementing translation policies [28]. Similar approaches have been taken across many jurisdictions around the world [29].

If using an AI-based tool, healthcare professionals generally need to ensure the translation provided is accurate and unlikely to cause harm to patients [30]. Using a professional language interpretation service can help limit the risk of liability for harm caused. If, however, a patient requires medical care and no formal language interpretation service is available, using language interpretation devices might be the only way to understand and address the patient’s needs. To minimize the risk of legal liability for harm caused, providers should make the kinds of decisions that another reasonable provider might make in similar circumstances and with similar resources [29]. Knowledge about the limitations of an AI-based application’s performance in the language used is therefore key to enabling physicians to make well-informed decisions.

### Data privacy and security considerations

As long as personal health information (PHI) collected is only that which is necessary to provide and support care, privacy legislation generally allows providers to collect and share PHI among treating healthcare workers (i.e. “the circle of care”) without the patient’s express consent. This implied consent exception also allows PHI to be used for particular tasks related to quality improvement (QI) at healthcare facilities [31]. However, third-party translation tools are commonly hosted on servers that are owned or leased by the company that created the tool. In the case of GT, this means that words will be spoken into the app, transmitted to some server where the language interpretation model resides, interpreted on that server, then transmitted back to the app interface. Unless the GT tool is not only local (i.e. stored on the user’s device), questions about data privacy, security and stewardship need to be addressed. Users who download these tools will be prompted via a user agreement to provide consent for the tool to process their data/inputs, but what the consent entails will have privacy implications that require assessment by legal experts. Differences in model architecture need to be accounted for in the specific legal analysis. However, for the before-mentioned reasons, physicians are sometimes forced to use language interpretation tools in the absence of comprehensive legal evaluations by their facility’s legal/privacy department. Data and security risks could be reduced by obtaining informed consent from the patient to use the device. Personal identifiers should still be avoided while using language interpretation tools. Many AI-based language interpretation tools like GT can, for a cost, be hosted on private cloud servers dedicated specifically to a healthcare institution, allowing for compliance with local privacy and laws and enabling compliant use of these tools.

## Discussion

This study evaluated the clinical performance of GT in non-urgent paediatric consultations and also reviewed its use from a legal/policy perspective. In the clinical performance test, the proportion of correctly interpreted paragraphs was high (83.5%-95.4%). It was key that speakers adhered to the official language pronunciation. The continuous, real-time check of the source language transcript and repetition of the paragraph if a mistake was detected increased the amount of correctly interpreted paragraphs by 2.8%-17.5% (Table 1, Fig. 2). Given the overall strong performance of GT with relatively few overall misinterpretations, healthcare institutions may begin to further explore adoption of AI-based translation tools as more readily available, easy to use, and cost effective means, particularly if professional live interpretation is not feasible. AI-based language apps may one day shift the power balance towards patients as they may bring these tools with them to their healthcare encounters, being less dependent on resources available within a healthcare institution. This potential can be very impactful however significant cautions need to be addressed by creating future health systems that mitigate the risk associated with tools like GT.

Performance of GT is best when users follow standard pronunciations and check the source language transcript for accuracy. This could lead to inequities. Minority caregivers with unique dialects, young patients not yet able to read and people with limited education may face more AI-interpretation errors. Similarly, those who are illiterate or neuro-divergent may experience increased misinterpretation risks. Health workers must recognize when AI-tools underperform and adapt to more suitable language interpretation methods.

Although in-person professional language interpretation is widely considered gold standard, previous research has demonstrated potential limitations of professional human interpreters in medical settings. One study revealed an average of 31 mistakes in medical visits requiring human-based interpretation, more than half of which significantly affected understanding [32]. Findings were worse for interpreters with less than 100 h of training [33]. While not providing cultural mediation or emotional understanding and support, GT may have the potential to have less errors than human-based interpretation services and perform with greater consistency if the above-mentioned precautions are taken. Given that single and seemingly simple interpretation mistakes can potentially cause serious harm to patients, further studies are needed to test GT’s performance in real-life contexts.

Both language interpretation strategies are time-consuming: administrative work to set up live interpretation takes time and limited availability of certain languages, particularly after office hours, can cause increased waiting times and delays in the delivery of care. While GT is readily available at no cost, repetitions of paragraphs with wrong source language transcripts are also time consuming and reduce the feasibility of language interpretation in clinical settings. A case-by-case approach, assessing advantages and disadvantages of both strategies, will help us find the best possible solution for each specific patient. For example, if presenting during a night shift, a first assessment with GT may help to initiate treatment. If during office hours, then the initial assessment can then be complemented by a conversation using live interpretation prior to discharge.

The legal/policy evaluation revealed a lack of defined gold standards for language interpretation in health contexts. The law however requires that consent for treatment be voluntary, informed, and given by a person who would be deemed legally competent [34]. This is only possible if the person can understand, respond and question the information provided. It seems reasonable to suggest that a positive right to interpretation services ought to exist, but Canadian lawmakers and many courts globally have not yet recognized such a right. Compelling criticism has also emerged suggesting that this ad hoc approach to funding/access might signal that policies of linguistic assimilation (whether intentional or inadvertent) are at play. This is a serious criticism beyond the scope of this analysis, but deserves greater attention by law/policy-makers. The right not to be discriminated against on the basis of the language one speaks can support a right to access interpretation services in healthcare. Supporting the growth of the medical interpretation industry will require policy-makers and insurers to consider implementing reimbursement policies so that the use of high-quality translation services is normalized as part of the standard of care [35].

This study has limitations. As performance of the device varied depending on the pronunciation of the speaker and background noise, reproducibility of the same quantitative results is limited, particularly as it was beyond the capacities of this study team to perform more than one performance test per language. However, the detailed step-by-step evaluation by an interdisciplinary team including medical and linguistic experts and a moderating third person ensured in-depth and multifaceted testing, likely to produce qualitative results that are generalizable to similar contexts. The use of a performance test methodology, validated by professional interpreters and pilot-tested and adapted by clinicians to their daily routine ensured the rigidity of the methodology and the relevance of the research to the real-life clinical setting.

Research, policy and practice implications: Political institutions should set the financial and legal frameworks needed to systematically ensure availability and funding of professional language interpretation services for patients with limited local language proficiency in health care settings. In addition, AI-based language interpretation has come a long way and is progressing quickly as large language models informing them also improve [22]. Findings of the performance test and of the legal and policy evaluation show that there is no reason to outright reject the use of these machine translation tools, particularly in the absence of professional language interpretation. Regulatory approval for the intended use of specific AI-based language interpretation tools should be sought and national data storage could increase data safety. Health care institutions should create institutional best practice guidelines for language interpretation to support clinicians in the provision of care to patients with limited language proficiency. Institutions need also to ensure that privacy and security risks have been accounted for. Health care providers could then apply these guidelines on a case-by-case approach and ensure best practices are followed when using AI-based language interpretation.

## Conclusions

Using GT for non-critical pediatric emergency consultations can improve communication when no interpreters are available, but requires careful execution, including verifying transcripts of source and target language, correct pronunciation, and distinguishing between literal and informal language. Establishing legal standards for interpretation in healthcare with appropriate funding is crucial. GT use should involve informed consent, avoid personal data, and use secure cloud solutions to reduce legal and data risks. This approach balances the benefits of machine translation with its limitations and ethical considerations, enhancing healthcare quality and respecting patient diversity.

## Supplementary Information

Supplementary Material 1.

## Data Availability

A summary of the data collected during the performance check is published as supplementary material. Additional material can be requested by the corresponding author via email.
